# Simulating block-scale flood inundation and streamflow using the WRF-Hydro model in the New York City metropolitan area

**DOI:** 10.1007/s11069-024-06597-y

**Published:** 2024-04-18

**Authors:** Berina Mina Kilicarslan, Marouane Temimi

**Affiliations:** Department of Civil, Environmental, and Ocean Engineering, Stevens Institute of Technology, Hoboken, USA

**Keywords:** Urban flooding, WRF-Hydro, Flood inundation, High resolution

## Abstract

This study assesses the performance of the Weather Research and Forecasting-Hydrological modeling system (WRF-Hydro) in the simulation of street-scale flood inundation. The case study is the Hackensack River Watershed in New Jersey, US, which is part of the operational Stevens Flood Advisory System (SFAS), a one-way coupled hydrodynamic-hydrologic system that currently uses the Hydrologic Engineering Center's Hydrologic Modeling System (HEC-HMS) to simulate streamflow. The performance of the 50-m gridded WRF-Hydro model was assessed for potential integration into the operational SFAS system. The model was calibrated with the dynamically dimensioned search algorithm using streamflow observations. The model performance was assessed using (i) streamflow observations, (ii) USGS HWMs, and (iii) crowdsourced data on street inundation. Results show that WRF-Hydro outperformed the HEC-HMS model. WRF-Hydro over and underestimated flood inundation extent due to the inaccuracy of the synthetic rating curves and the modeling structure errors. An agreement was noticed between WRF-Hydro and crowdsourced data on flood extent.

## Introduction

Urban watersheds especially those with a high impervious percentage (Chen et al. 2015a, b) and those subject to changing climatic conditions that generate more intensified extreme rainfall events (O’Donnell and Thorne 2020) are highly susceptible to urban flooding. Due to combinations of these anthropogenic and natural factors, urban flooding as a result of extreme weather events may lead to significant economic damages and continue to put increasing pressure on communities (Hettiarachchi et al. 2018). Therefore, it is crucial to create accurate hydrologic and hydraulic models in order to simulate flood inundation effectively and determine the possible effects of extreme events.

The Hackensack River watershed, the primary focus of this study, is located within the New York City metropolitan area, at the border between northern New Jersey and southern New York. The watershed is densely populated and flood-prone while being part of the most highly developed floodplain with economic assets along the East Coast of United States (Saleh et al. 2017). Throughout the years, the region has been severely impacted by several extreme weather events, including hurricanes like Hurricanes Irene and Sandy, in 2011 and 2012, respectively. The development of a decision support system that generates an operational forecast of the impact of major weather events and assesses their damage is crucial for densely populated watersheds like the Hackensack River.

Stevens Institute of Technology developed the Stevens Flood Advisory System (SFAS) specifically for operational flood inundation mapping in the New York City metropolitan area (Georgas et al. 2016). This operational framework integrates a 3D hydrodynamic model with the hydrological and storm surge models. The inland hydrology component of the SFAS system relies on a lumped hydrological model, namely, the Hydrologic Engineering Center's Hydrologic Modeling System (HEC-HMS) (Saleh et al. 2016, 2017), which was calibrated over the Hudson River watershed region, encompasing the Hackensack River watershed. The model comprises all the main tributaries and reservoirs and uses gridded meteorological forcing. The HEC-HMS model has been widely used across numerous modeling frameworks (Unduche et al. 2018; Wijayarathne and Coulibaly 2020; Mourato et al. 2021).

More recently, data-driven models were also investigated to simulate streamflow in the Hackensack watershed during significant flood events. Tounsi et al., (2022) used a long short-term memory (LSTM) model to capture the reservoir management rules in the Hackensack River watershed and to forecast the streamflow values at the watershed downstream station. Data-driven models were also successfully tested in the simulation of streamflow at a continental scale. Using the Catchment Attributes and Meteorology for Large-sample Studies dataset (CAMELS), the LSTM model has been proven efficient in simulating streamflow (Lees et al. 2021). Several other data-driven methods were tested at the watershed scale and proven to be reliable (Chen et al. 2015a, b; Hu et al. 2018; Jiang et al. 2022; Kratzert et al. 2018).

The limitation of lumped and data-driven models consists of their non-suitability for the simulation of distributed flood inundation within the watershed. Instead of capturing the spatial heterogeneity of the hydrological processes, lumped models average the hydrological states over the whole watershed and simulate streamflow at specific river stations that encompass the hydrologic response over the entire upslope area as it is the case with the HEC-HMS model in the SFAS system (Saleh et al. 2016). The data-driven models join the lumped ones in their incapabilities to explicitly represent the processes contributing to the watershed hydrologic response and their spatial distribution. Consequently, it is difficult to capture the complexity of systems like urban watersheds and accurately predict the potential effect of extreme weather events at particular sites. Both model categories are unable to simulate the spatial distribution of the hydrologic response in the watershed. These models are usually trained, in the case of data-driven models, or calibrated, in the case of lumped ones, to simulate streamflow at specific forecast points rather than determining which areas, e.g, block or street intersection, in the watershed are more impacted by an event so that intervention and possible evacuations can be planned properly.

The advent of gridded models like the Weather Research and Forecasting Model Hydrological modeling system (WRF-Hydro) (Gochis et al. 2020) has certainly helped in the development of models that can simulate the spatial distribution of the hydrologic and hydraulic processes within the watershed. This is a significant improvement over lumped models for the cases that the gridded information is crucial to accurately assess the impact of extreme events. The model was used by Viterbo et al. (2020) to simulate the impact of a flash flood in Ellicott City, in May 2018. Smith et al. (2021) evaluated the two different flood modeling systems, specifically WRF-Hydro and ADHydro for predicting urban flooding in hyper-resolution over Charlotte, NC municipal areas. Additionally, Kim et al. (2021) assessed the impact of the resolution of urban scale flood modeling by using WRF-Hydro for three highly urbanized catchments over the Dallas-Fort Worth metropolitan area, TX. Results indicate that the fully distributed modeling system that utilizes WRF-Hydro can accurately simulate the hydrological response to intense rainfall event. They attribute the common source of uncertainty in the WRF-Hydro model to the quality of the forcings, notably its temporal and spatial resolution. Often a mismatch is reported between the scale of the surface routing scheme, usually in the order of 100 m, and the meteorological forcing that can be as coarse as a few tens of kilometers. The complexity introduced to the model as a result of their high resolution and the large extent of the watersheds to model in metropolitan areas is also stated as additional sources of errors in application over urbanized watersheds (Viterbo et al. 2020; Kim et al. 2021). Predicting flood inundation in urban watersheds requires small-scale simulation and therefore high-resolution models which tend to be sensitive to the quality of the forcing and the used parametrization. There has been growing interest in the development of watershed-scale models using WRF-Hydro which can seamlessly simulate hydrological and hydraulic processes at a spatial scale that is appropriate for the analysis of the impact of extreme events, especially in densely populated watersheds, like the Hackensack River one that is investigated in this study. In this regard, the WRF-Hydro model is considered a perfect candidate.

The goal of this study is to assess a new gridded WRF-Hydro-based model that simulates streamflow and flood inundation in the Hackensack River Watershed in New Jersey. The model is a candidate for deployment in the operational Stevens Flood Advisory System. The SFAS relies in its current version on HEC-HMS to simulate hydrological processes and uses the outflow to force HEC-RAS and coastal models. In several studies, WRF-Hydro is also utilized and suggested as an upstream boundary condition for the HEC-RAS model in the absence of stream gauge records on the upstream side of watersheds (Papaioannou et al. 2019; Alipour et al. 2022). The proposed WRF-Hydro model features a block-scale resolution of 50 m for the channel routing scheme and 200 m for the surface overland routing scheme. The model is expected to offer an improvement over the existing hydrologic component in the SFAS system which is a novel contribution to an essential flood alert system that is used in the New York City metropolitan area. The case study is selected as Hurricane Irene which was recorded in August 2011. The goal is to assess WRF-Hydro with respect to in situ observations, the operational version of HEC-HMS, and crowdsourced information in the aftermath of the event. The novelty of this study consists of simulating hyper-resolution flood inundation in an urban environment with a controlled reservoir using WRF-Hydro. It demonstrates the capability of WRF-Hydro in simulating processes at this high spatial resolution while accounting for the behavior of reservoirs. In addition, this study demonstrates the feasibility of the potential integration of WRF-Hydro in an operational regional flood advisory system like SFAS which could serve as a case study for other implementations in similar urban contexts.

## Methodology

### Study area

**Fig. 1:**
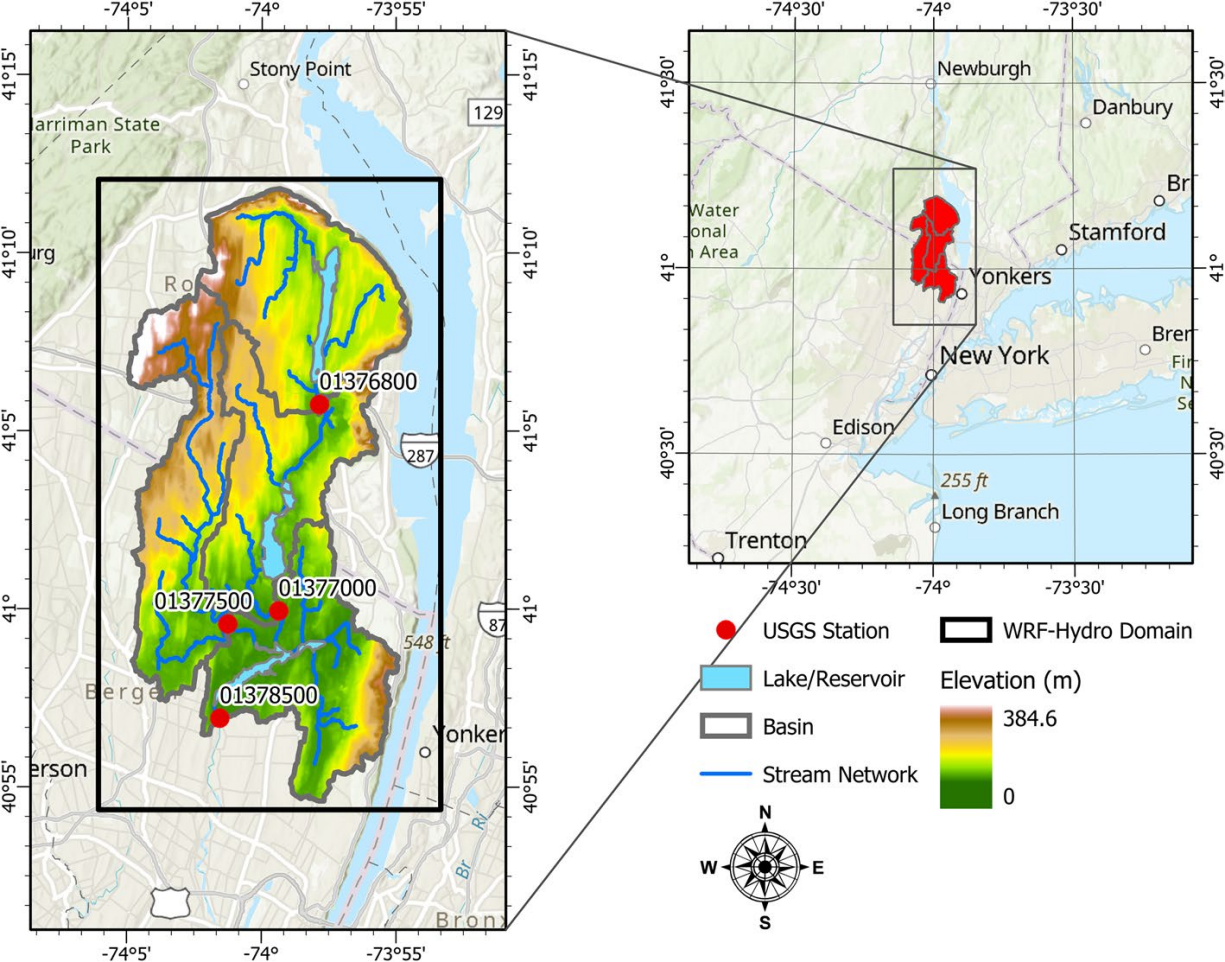
The Hackensack River watershed in New Jersey

The case study is the Hackensack River watershed which is located in northeastern New Jersey and stretches beyond the New Jersey-New York borders (Fig. 1). Its northern upstream limits start in Rockland County, New York, from Lucille and Deforest Lakes, and then flow all the way downstream to Newark Bay. The watershed is densely urbanized and includes critical economic assets and important reservoirs and lakes. Over 1 million residents in the New York City metropolitan area receive ~ 120 million gallons of drinking water every day from the reservoirs within the watershed, namely, Woodcliff Lake Reservoir, Lake Tappan, Lake Deforest, and the Oradell Reservoir (State of New Jersey Department of Environmental Protection 2017). The downstream section of the watershed towards Newark Bay is subjected to the tidal effect of Newark Bay which propagates all the way upstream to the level of New Milford USGS station on the Hackensack River. Since this study investigates only the combination of riverine and surface flooding, the part of the watershed downstream of New Millbrook station was excluded from the studied domain (Fig. 1). The drainage area of the studied part of the basin is around 294 km^2^. The northwest part of the watershed shows a steep slope characteristic with an altitude reaching up to ~ 385 m. Based on the National Land Cover Database (NLCD) 2011, the predominant land cover types over the region are defined as Developed and Deciduous Forest categories.

### Event synoptics

**Fig. 2:**
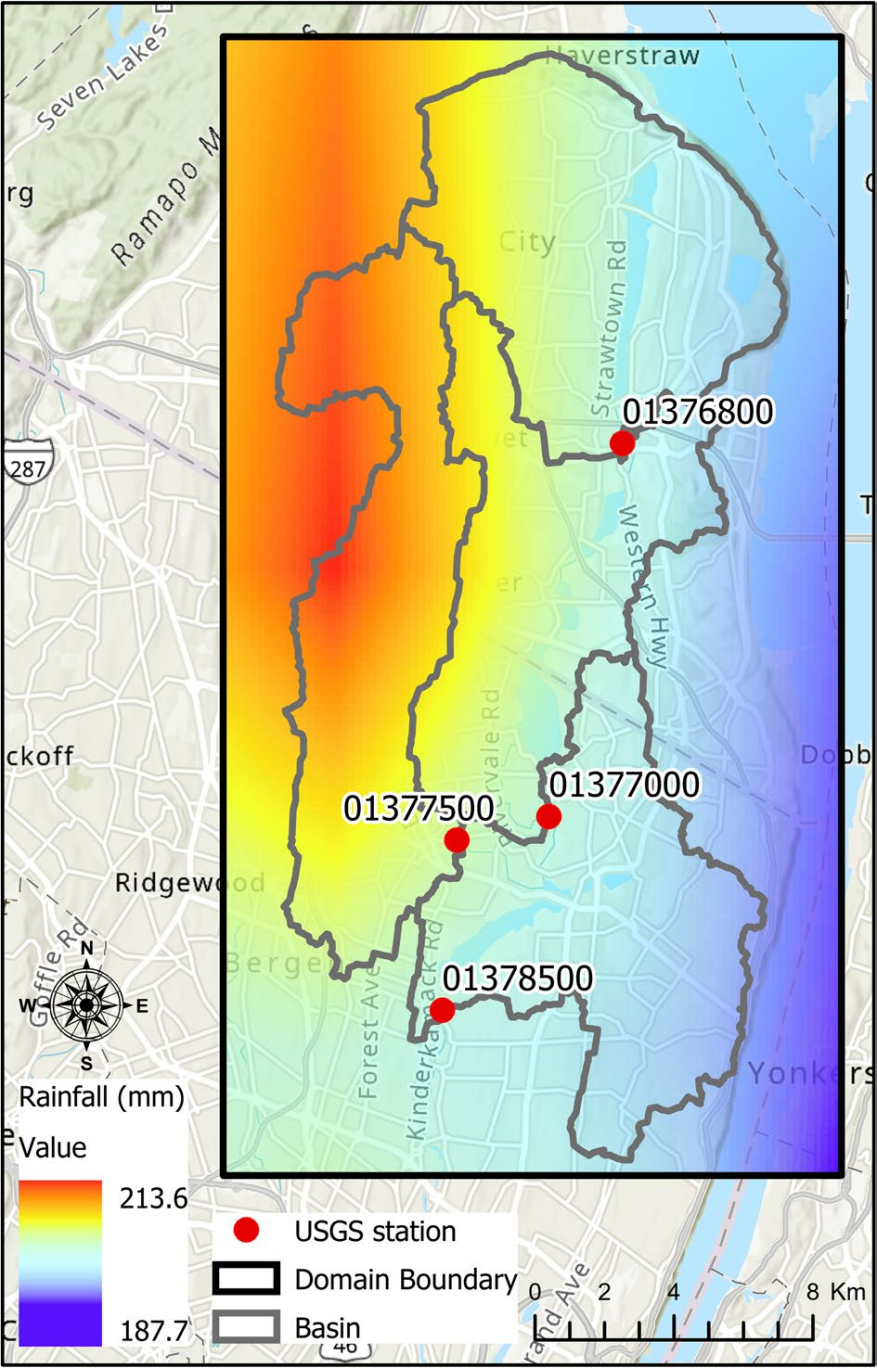
Spatial distribution of cumulative rainfall (mm) retrieved from NLDAS-2 dataset over Hackensack basin during Hurricane Irene

In August 2011, Hurricane Irene caused extensive property damage in the New York City metropolitan area, including the Hackensack River Watershed in New Jersey. After the first landfall in North Carolina on August 27, 2011 morning, the hurricane crossed again the ocean and made another landfall in southeastern New Jersey, on August 28, 2011, at 05:35 A.M. Since 1903, this is the first time a hurricane has made landfall in New Jersey. The total precipitation records range from 6.5 to 8.6 inches in the Passaic and Hackensack River Basins. The significant rainfall received during August in New Jersey made it the wettest month on record since 1895. Therefore, the antecedent wet conditions in the state also had a significant impact on the severity of the flooding after the event. Many stream gauges flooded over the state including those located in the Hackensack River watershed were severely impacted which led to streamflow records higher than a 100-year return period (2011a). Figure 2 shows the spatial distribution of cumulative rainfall across the Hackensack basin derived from the North American Land Data Assimilation System Phase-2 (NLDAS-2) dataset between August 27, 2011, 04:00 P.M. and August 28, 2011, 04:00 P.M. The cumulative precipitation varies between 188 and 214 mm (7.4–8.4 inches) over 25 h. Mostly, the western side of the basin received the highest rainfall amount. This implies that tributaries like the Pascack Brook (USGS station 01377500) that drains the western part of the watershed to the Hackensack River are expected to experience significant stress during the event. This will be verified further in the results section by analyzing water level and flood inundation at the station.

### Datasets

The NLDAS-2 dataset is utilized as the forcing dataset for the model simulations (Cosgrove et al. 2003). Both the precipitation and the other atmospheric forcings were derived from the NASA NLDAS-2 website. The dataset is downscaled from 0.125° × 0.125° to a 200-m resolution using the bilinear interpolation technique, which is the resolution specified for the land surface model (LSM) in the WRF-Hydro model. The downscaling of the atmospheric forcing is necessary to match the resolution of the land surface model, namely, Noah-MP. The terrain-based parameters and routing stack in 50-m resolution for WRF-Hydro are generated based on the USGS 1/3 arc-second (10-m) Digital Elevation Model (DEM) product by using the WRF-Hydro Pre-Processing ArcGIS toolbox (Sampson and Gochis 2015). The Moderate Resolution Imaging Spectroradiometer (MODIS) Modified IGBP 20-category landcover product with 500-m spatial resolution provides land cover classifications for the LSM, while the soil classifications are produced from the 1-km STATSGO database.

For the evaluation of the simulated streamflow, the following USGS stations of the Hackensack River at West Nyack NY (01376800), Hackensack River at Rivervale NJ (01377000), Pascack Brook at Westwood NJ (01375000), and Hackensack River at New Milford NJ (01378500) are considered (Fig. 1). The water depth predicted by WRF-Hydro is evaluated using surveyed USGS high water mark (HWM) data (Watson et al. 2013). In addition, a qualitative assessment of the simulated water depth or flood inundation mapping is made based on the crowdsourced data. To this end, pictures released in local media in the aftermath of Hurricane Irene were geotagged manually. The surface water depth output from the WRF-Hydro simulation is overlaid on the Google Earth 3D map layer and visually compared with the geotagged pictures from the same angle that the picture was captured.

### Model description

A fully distributed, multi-physics model called Weather Research and Forecast Model Hydrological Extension (WRF-Hydro version 5.1.1) developed by the National Center for Atmospheric Research (NCAR) is used in this study (Gochis et al. 2020). WRF-Hydro modeling system relies on a 1-D column land surface model (LSM) called Noah-MP, which is responsible for the energy and moisture fluxes in a vertical direction and water balance calculation in the soil column. After the moisture state calculations are performed, water is partitioned from LSM grids into the integer portion of the high-resolution routing grids. Routing processes are represented in higher resolution compared to LSM grid resolution. A high-resolution routing stack is created by disaggregating the 200-m LSM grids into 50-m high-resolution grids for overland and channel routing schemes. Thereafter, the lateral redistribution of the water is performed based on the different routing modules (overland, subsurface, channel, and reservoir routing). Following the execution of the routing modules, the fine-grid values are aggregated back to the native land surface model grid, and the soil moisture is redistributed through the soil column.

Diffusive wave formulation is adopted for the overland and channel flow routing schemes (Gochis et al. 2020). For the overland flow, the two-dimensional continuity equation is

1 $$\frac{\partial h}{{\partial t}} + \frac{{\partial q_{x} }}{\partial x} + \frac{{\partial q_{y} }}{\partial y} = i_{e}$$

where *t* is time, *h* is is the surface flow depth; *q*_*x*_ and *q*_*y*_ are the unit discharges in the x and y directions, respectively; and *i*_*e*_ is the infiltration excess. Manning`s equation is used to calculate the *q*_*x*_ and *q*_*y*_. For the x direction;

2 $$q_{x} = \alpha_{x} h^{\beta }$$

where *β*-unit dependent coefficient equals 5/3, and

3 $$\alpha_{x} = \frac{{S_{fx}^{1/2} }}{{n_{ov } }}$$

where *n*_*ov*_ is the tunable roughness coefficient for land surface. The momentum equation used in diffusive wave for x dimension is

4 $$S_{fx} = S_{ox} - \frac{\partial h}{{\partial x}}$$

where *S*_*fx*_ is the slope of the energy grade line, and *S*_*ox*_ is the terrain slope in x direction.

Channel routing is performed using an explicit, one-dimensional, variable time-stepping diffusive wave formulation. Similarly, the continuity equation for the diffusive-wave formulation is given as

5 $$\frac{\partial A}{{\partial t}} + \frac{\partial Q}{{\partial x}} = q_{lat}$$

where *q*_*lat*_ is the lateral inflow rate into the channel. The momentum equation is given as

6 $$\frac{\partial Q}{{\partial t}} + \frac{{\partial \left( {\beta Q^{2} /A} \right)}}{\partial x} + gA\frac{\partial Z}{{\partial x}} = - fAS_{f}$$

where *x* is the streamwise coordinate, *A* is the flow area of cross-section, *β* momentum correction coefficient, *Z* is the water surface elevation, *g* is the gravity and *S*_*f*_ is the friction slope given as

7 $$S_{f} = \left( \frac{Q}{K} \right)^{2}$$

where *K* is the conveyance computed from Manning`s equation:

8 $$K = \frac{{C_{m} }}{n}AR^{2/3}$$

where *n* is Manning`s roughness coefficient for the channel, *C*_*m*_ is a dimensional constant and *R* is the hydraulic radius.

Diffusive-wave formulation enables the model to calculate the backwater effect through the adverse slope for overland flow. However, the backwater effect accounting for the overbank flow from the channel to the terrain cannot be computed in the existing channel routing scheme. Therefore, to account for the riverine flooding over terrain, the Height Above the Nearest Drainage (HAND) method is adopted for the inundation depth and extent calculations. Using this approach, the map layer of vertical distance from any location on the terrain to the river surface is generated. HAND layers are calculated based on the USGS 10-m DEM which is interpolated to the 50-m resolution to match the WRF-Hydro high-resolution routing grids. River stage values for the WRF-Hydro streamflow outputs are generated by using Synthetic Rating Curves (SRC) defined CONUS-wide for each National Water Model (NWM) reach. Given that the NWM reaches are defined based on the National Hydrography Dataset Plus (NHDPlus) V2.1 dataset, the sub-watersheds corresponding to each reach were used as the border to clip the channel grid outputs storing streamflow values of WRF-Hydro. The simulated peak streamflow values within these sub-watersheds are extracted and utilized to determine the peak stage values by using SRCs. SRCs are obtained from the National Flood Interoperability Experiment (NFIE) repository. Three major reservoirs (Lake Oradell, Lake Tappan, and Lake DeForest) in the study area are defined in the model. For each reservoir, both weir and orifice structures are defined, and the outflow calculations are performed based on the simple level pool routing scheme (Gochis et al. 2020). The model is compiled on LINUX Ubuntu with a one-way coupling option, utilizing 12 CPUs for the simulations.

WRF-Hydro model is tested in this study for an eventual implementation in the SFAS (Jordi et al. 2019). SFAS is an operational flood forecasting and warning tool that encompasses the New York City metropolitan area. It includes coastal, hydrologic, and hydraulic models that are forced with an ensemble of nearly 100 members from different sources such as the Global Ensemble Forecast System (GEFS) and European Centre for Medium-Range Weather Forecasts (ECMWF). Additionally, the modeling system integrates the inland hydrology mechanisms into the coastal model as a boundary condition. By incorporating river flows, tides, and storm surges, it provides more accurate results compared to the other existing coastal models which lack inland hydrology. The hydrologic model used in the SFAS is HEC-HMS covering several watersheds in the region including the Hackensack River Watershed. The model utilizes lumped parametrization approach and implements the SCS-Curve Number method (Saleh et al. 2016) for runoff calculations. It comprises all the reservoirs within the watershed and captures their operations.

### Model evaluation and performance metrics

The model is assessed based on a variety of outputs, including streamflow, flood inundation depth, and extent. Comparison between the observed or benchmark and the simulated data is performed based on different model performance metrics described below.

Regarding the comparison between the observed and the simulated hydrographs, Root Mean Square Error (RMSE), Nash–Sutcliffe Efficiency (NSE), and Kling-Gupta Efficiency (KGE) are sensitive to both the temporal and the volumetric distribution in the hydrograph and indicate the overall model fit. RMSE (Eq. 9) calculates the standard deviation of the residual between the observed and the simulated data. The residuals are squared prior to being averaged which helps to see the large errors in the simulated hydrograph. NSE (Eq. 10) measures the variance of the residual between observation and the simulation to the variance of the observation. Aside from the low weight of the bias component in NSE, KGE (Eq. 11) gives equal weight to bias (β), correlation (r), and standard deviation (γ) components. These metrics are calculated based on the hourly time step same as the model time step. Formulas of mentioned metrics are given as follows:

9 $$RMSE = \sqrt {\frac{{\mathop \sum \nolimits_{i = 1}^{N} \left( {x_{obs}^{i} - x_{sim}^{i} } \right)^{2} }}{N}}$$

10 $$NSE = 1 - \frac{{\mathop \sum \nolimits_{i = 1}^{N} \left( {x_{obs}^{i} - x_{sim}^{i} } \right)^{2} { }}}{{\mathop \sum \nolimits_{i = 1}^{N} \left( {x_{obs}^{i} - \overline{x}_{obs} } \right)^{2} }}$$

11 $$KGE = 1 - \sqrt {\left( {r - 1} \right)^{2} + \left( {\beta - 1} \right)^{2} + \left( {\gamma - 1} \right)^{2} }$$

$$\beta = \frac{{\mu_{sim} }}{{\mu_{obs} }}$$

$$\gamma = \frac{{{\raise0.7ex\hbox{${\sigma_{sim} }$} \!\mathord{\left/ {\vphantom {{\sigma_{sim} } {\mu_{sim} }}}\right.\kern-0pt} \!\lower0.7ex\hbox{${\mu_{sim} }$}}}}{{{\raise0.7ex\hbox{${\sigma_{obs} }$} \!\mathord{\left/ {\vphantom {{\sigma_{obs} } {\mu_{obs} }}}\right.\kern-0pt} \!\lower0.7ex\hbox{${\mu_{obs} }$}}}}$$

where $N$ is the total number of the time steps for hydrograph evaluations or the number of HWMs for the water level comparison. $x_{obs}$ and $x_{sim}$ stands for observed and simulated data at $i$th time step or HWM location. The mean of observed and simulated data is given by $\mu_{obs}$ and $\mu_{sim}$, while the standard deviation of observed and simulated data is given by $\sigma_{obs}$ and $\sigma_{sim}$.

Percent Bias (PBias) is used to determine the average error between the observed and the simulated data (Eq. 12). In terms of hydrograph interpretation, it gives information about how accurately the model can simulate the water balance.

12 $$PBias = { }\frac{{\mathop \sum \nolimits_{i = 1}^{N} (x_{sim}^{i} - x_{obs}^{i} )}}{{\mathop \sum \nolimits_{i = 1}^{N} (x_{obs}^{i} )}}{ }x{ }100$$

In addition to Bias and RMSE, Mean Absolute Error (MAE) is calculated to see the average residual for the evaluation of the simulated water level compared to the USGS HWMs (Eq. 13).

13 $$MAE = { }\frac{{\mathop \sum \nolimits_{i = 1}^{N} |x_{sim}^{i} - x_{obs}^{i} |}}{N}$$

The flood inundation extent comparison is performed based on the peak water depth simulated over the sub-catchment and the corresponding surface area of the inundation.

### Model calibration

In this section, it is aimed to calibrate the WRF-Hydro model using an event of similar magnitude as Hurricane Irene, recorded before or after the case study. In this context, superstorm Ida which struck the Gulf region and progressed all the way to the northeast region as a heavy storm was identified as an ideal candidate for conducting the model calibration.

Various objective functions were tested in the calibration process, including combinations of objective functions, within a two-stage calibration process to assess their impact on the calibration results. The second calibration stage is expected to build on the achievements of the first calibration and improve it. It was determined that the combination of KGE-RMSE is the one that leads to the best agreement between observed and simulated streamflow. The combination of objective functions consists of running a first two-hundred-iteration calibration with the KGE objective function. Then, the obtained parameters are used in a second calibration stage with the same number of iterations utilizing the RMSE as the objective function.

In this study, the Dynamically Dimensioned Search (DDS) Algorithm developed by  Tolson and Shoemaker (2007) is used. The DDS algorithm begins with the global search for the selection of the parameter set and then it converges to a local solution by dynamically and probabilistically reducing the size of the parameter set perturbed in each iteration. The degree of the perturbation of the selected parameters is calculated based on the normal distribution aiming to converge to an optimal solution based on the user-defined number of iterations. This algorithm is particularly well-suited for complex and distributed models that need longer simulation times compared to other hydrological models. Therefore, it is commonly utilized for the WRF-Hydro model having such complex characteristics and long iteration times.

The calibrated model parameter set with their ranges is given in .

Table 1. All parameters are unitless and defined as a factor for the variables in the model. In this calibration procedure, it was intended to include the parameters commonly suggested in previous studies having an impact on the soil properties and the routing process (Yucel et al. 2015; Silver et al. 2017; Mehboob et al. 2022). The parameters were selected based on two groups of physical significance on the simulated hydrograph as described in Kilicarslan et al. (2021): the ones controlling hydrograph volume and shape (temporal distribution and peak time timing).

**Table 1:** Calibrated parameter set based on their physical significance in the model parametrization

Major control on the hydrograph	Parameter	Name	Range
Volume	REFKDT	Infiltration factor	0.5–5.0
	RETDEPRTFAC	Surface retention depth factor	0.0–10.0
	SLOPE	Deep drainage coefficient	0.1–1.0
Shape (temporal distribution and peak timing)	OVROUGHTRTFAC	Surface roughness coefficient factor	0.1–1.0
	MANN	Manning roughness scaling factor	0.5–2.0
	LKSATFAC	Saturated hydraulic conductivity factor	10–10,000

The infiltration factor (REFKDT) controls the volume of extra-filtration on each grid cell calculated after the rainfall is infiltrated and saturated the soil column. It is one of the parameters that have a significant impact on hydrograph volume. Other parameters that influence the hydrograph volume include surface retention depth factor (RETDEPRTFAC) and deep drainage coefficient (SLOPE) which are responsible for the amount of water that is ponded over each grid cell prior to contributing to runoff and the openness of the bottom soil column. The surface roughness coefficient factor (OVROUGHRTFAC) and Manning’s coefficients factor (MannN) have a significant impact on the surface and channel routing schemes of the model, respectively. Reducing the OVROUGHRTFAC value accelerates the surface runoff whereas reducing the MannN shortens the conveyance time of the water throughout the channel. Lastly, the lateral saturated hydraulic conductivity factor (LKSATFAC), which affects the lateral distribution of the water between adjacent soil columns, influences both the volume of the hydrograph and its distribution over the time series.

The calibration was performed using streamflow observations at New Milford station, which is the most downstream station in the watershed, despite the presence of other USGS stations within the watershed. The configuration of the used DDS algorithm allows for the consideration of one single station. The adjusted parameters resulting from the calibration will apply to the entire watershed. Future versions of the algorithm with the capability of including several stations and generating different parameter sets for different sub-watersheds have potential to reduce the uncertainty introduced by the homogeneity of the parameter sets imposed by the single station-based calibration.

## Results and discussion

### Calibration & validation of WRF-Hydro

**Fig. 3:**
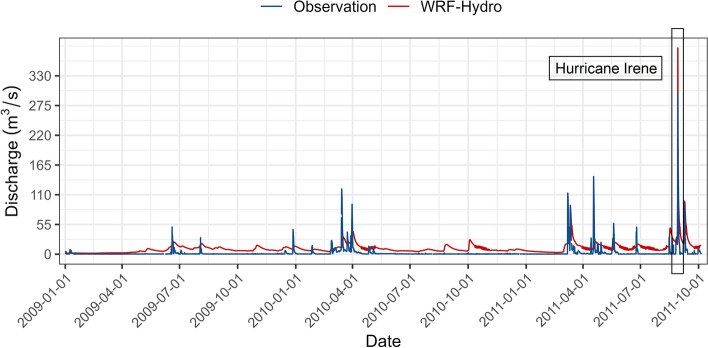
WRF-Hydro spin-up simulation (red) and the recorded (blue) hydrograph at New Milford station for Hurricane Irene

Figure 3 shows the performance of the model at New Milford station during the spin-up simulation which covered approximately 2 years, ending on October 1st, 2011. The spin-up period comprised various events including the ones with high streamflow values like those in April 2010 and April and May 2011. Overall, the model simulated peaks properly and responded accordingly to extreme precipitation events. However, an overestimation of the observed streamflow was noticed during some events, for instance, in September and October 2010. This could be attributed to the uncertainty of the meteorological forcing or the absence of reservoir management rules upstream of New Milford that retained the runoff in the Oradell reservoir, and which are not implemented in the current version of the model. In addition, the spin-up simulation shows that the model has some deficiencies in simulating baseflow properly. Throughout the spin-up period, the simulated baseflow was systematically lower than the observed one. It is expected that the calibration process addresses this flaw. Nevertheless, the spin-up simulation remains necessary to prepare the model and generate realistic, yet not necessarily accurate, values for streamflow, surface, and groundwater variables. The spin-up simulation generates the necessary restart files for a warm restart of the model after its calibration. The state variables obtained from the spin-up simulation are used as the initial condition for the model.

The calibration can be conducted using events, preferably of the same magnitude as the study case, which is Hurricane Irene, regardless of the date of their occurrence prior to or after Irene. Hurricane Irene was the last extreme event of such magnitude that impacted the region. In this study, other extreme events, yet not hurricanes, like the major storm Ida that impacted the watershed and the region in early September 2021 were used to conduct the calibration of the model using the DDS algorithm. The calibration of the WRF-Hydro model was carried out with the warm start setup after running a two-year-long spin-up simulation prior to storm Ida. The restart files used to initialize the calibration simulations were obtained from the spin-up simulation. The calibration is performed for the USGS streamflow observation at New Milford station.

Table 2 includes the default and the calibrated model parameter sets. After the calibration the REFKDT value was increased from 3 to 4.68 to reduce surface runoff volume that is contributing to the streamflow. RETDEPRTFAC increased from 1.0 to 7.1 mm, indicating that water levels exceeding 7.1 mm will be diverted as runoff. SLOPE value remains constant at 0.1. Both OVROUGHRTFAC and MannN are decreased to 0.4 and 0.9, respectively, to meet the lag time between the recorded and the simulated hydrograph peaks. The decrease in these two parameters leads to accelerated runoff and streamflow respectively. The calibration reduced the lateral hydraulic conductivity factor value-LKSATFAC from 1000 to ~ 220 (Table 2). Lower LKSATFAC restricts the movement of the lateral subsurface flow, and results in slower subsurface flow.

**Table 2:** Default and calibrated model parameters

	REFKDT	RETDEPRTFAC	SLOPE	OVROUGHRTFAC	MAnnN	LKSATFAC
Default	3.0	1.0	0.1	1.0	1.0	1000
Calibrated	4.68	7.14	0.1	0.42	0.90	220.42

**Fig. 4:**
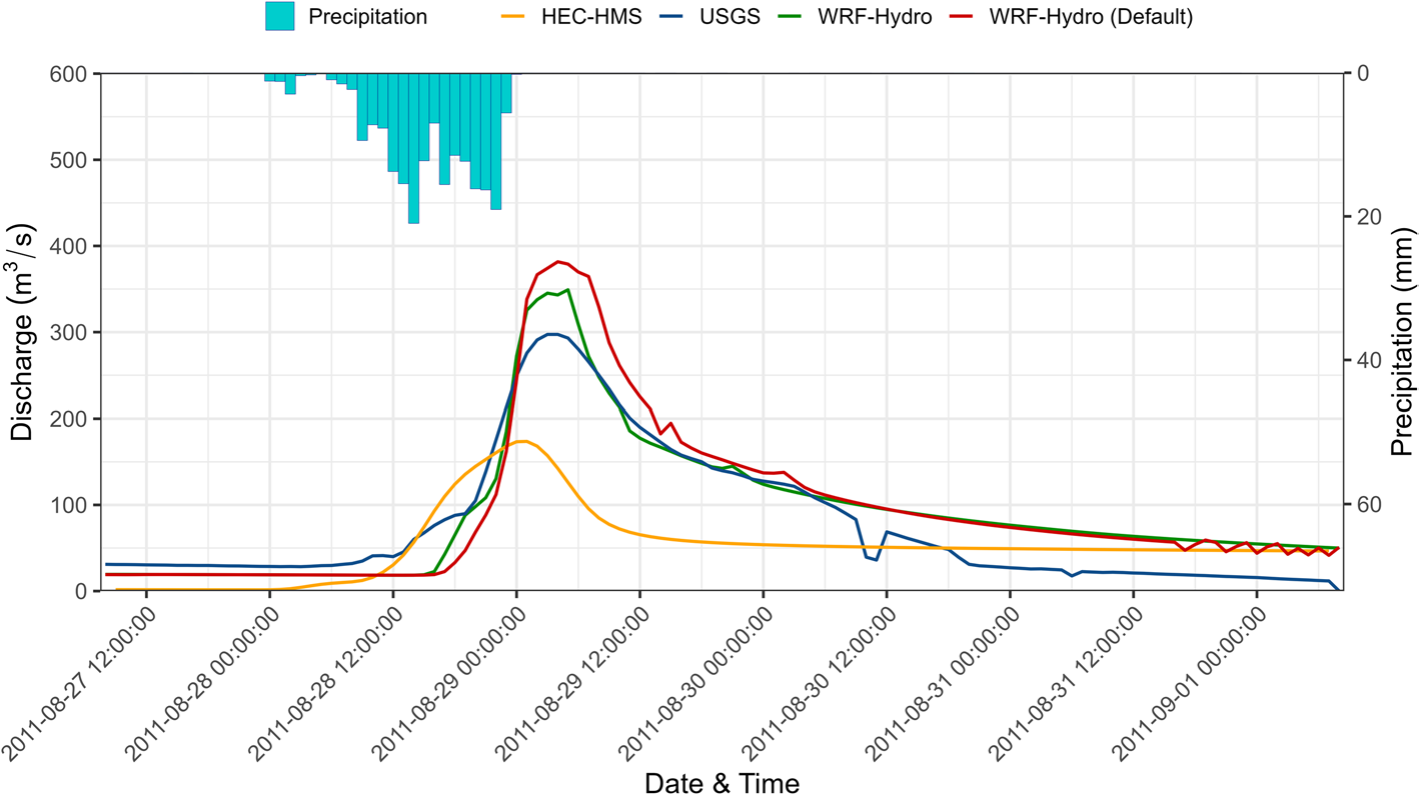
Evaluation HEC-HMS and WRF-Hydro streamflow simulations during Hurricane Irene at New Milford station (01378500)

The validation of the model is performed using the reference event, Hurricane Irene (08/27–09/01/2011). Figure 4 compares streamflow observations at New Milford to those simulated with WRF-Hydro using the default parameter set and the calibrated one presented in Table 2. For the default simulation, there is an observed tendency to overestimate the volume during peak and recession periods. The overestimation at the peak time and the falling limb are reduced with the calibrated parameter set. The simulation started on the 27th of August, a day prior to the start of the event. The start of the simulation shows that both configurations led to an underestimation of the baseflow prior to Hurricane Irene. However, the discrepancy is in the order of 10 m^3^/s and should not significantly impact the estimation of the event streamflow which reaches values in the order of 300 m^3^/s. The start of the increase in the observed streamflow was on the 28th of August around 1 am, about 12 h after the start of the rainfall which is indicative of the watershed lag time. This value is similar to the one reported by Tounsi et al. (2022) who used an LSTM model to infer reservoir rules in the same watershed, the Hackensack River watershed. Tounsi et al. (2022)conducted a feature analysis and found that a 12-h lead time is the most influential of streamflow estimates at New Milford. The simulated hydrographs showed a delayed increase of streamflow at the start of the event, yet the increase was steeper with the calibrated configuration of the model compared to the default one. This could be attributed to various reasons like reservoir management rules and excessive retention of water in the watershed lakes in the model. The misrepresentation of the hydrological processes, namely surface roughness and an overestimation of the infiltration could also lead to such delay. One should note that reservoir operations, represented in the model with a weir and an orifice were not included in the calibration set. The inference of reservoir management rules through the calibration process that involves streamflow observations only downstream of the reservoir is not straightforward. Tounsi et al. (2022) demonstrated that the inference of such rules requires a multi-year analysis of streamflow observations upstream and downstream of their reservoir. In addition, the DDS algorithm in its current version can only account for one station which is the New Milford one downstream of the reservoir. This can be improved in future work where the DDS-based calibration can involve multiple stations and adjust iteratively and simultaneously the parameters of various sub-watersheds within the study domain.

The calibrated version of the model showed a reduction in the estimation of the peak flow to a closer value to the observed one compared to the default simulation (Fig. 4). The timing of the peak flow was correctly simulated by the default and calibrated versions when compared to observations. The value of the peak flow was reduced from 382 to 349 m^3^/s. Then, the descending limb of the hydrographs showed a better agreement again with the calibrated version of the model which started the decline of streamflow earlier than the default version of the model. Towards the end of the event, early on the 30th of August, both versions, the default and the calibrated departed from the observations and overestimated streamflow corroborating the limitations of the model to properly simulate baseflow as it was noticed prior to the start of the event.

In addition, Fig. 4 displays the simulated streamflow at New Milford using the operational HEC-HMS version of the SFAS system. For comparison purposes, the HEC-HMS model was forced using the same meteorological data used with WRF-Hydro, namely, the gridded NLDAS-2 dataset. Figure 4 shows an underestimation of streamflow at New Milford with HEC-HMS. The peak time also occurs earlier compared to the observed one. Overall, Fig. 4 shows that a better performance is obtained with a calibrated version of WRF-Hydro. This can be corroborated with a quantitative assessment as reported in Table 3.

**Table 3:** HEC-HMS, WRF-Hydro default, and validation statistics calculated for Hurricane Irene at New Milford station (01378500)

	Corr	PBias	RMSE	KGE	NSE
WRF-Hydro-default	0.93	22.01	37.95	0.71	0.76
WRF-Hydro-validation	0.93	16.81	31.53	0.82	0.84
HEC-HMS	0.72	− 32.47	61.86	0.36	0.38

Table 3 summarizes the calculated metrics for the simulations with the default and the calibrated parameter sets. According to the table, the improvement obtained with the validation event is noticeable. Metrics including KGE and NSE, particularly measuring the overall performance are calculated as ~ 0.8 which demonstrates that the model effectively captures the event. The RMSE value, another performance metric showing the average total error in the simulated hydrograph, was reduced by ~ 17% also depicts that the overall model performance is improved. The PBias showed a reduction of about 24%. The correlation value remained the same, i.e. 0.93 when the calibrated version of the model was used. This indicates that both models were able to capture the covariance properly. This can be corroborated by the reported agreement on the timing of the peak flow. Nevertheless, the calibrated version of the model clearly introduced an improvement by reducing the RMSE and Pbias and increasing the KGE and NSE.

One should note that the benchmark performance here is the simulation of Hurricane Irene in 2011 using the operational HEC-HMS-based flood advisory system which was described in Saleh et al. (2017). Table 3 reports the performance metrics obtained with the operational HEC-HMS version of the SFAS system and compares them to those obtained with the default and calibrated versions of WRF-Hydro. The HEC-HMS model misses the peak time and volume in the observed hydrograph as shown in Fig. 4. The correlation coefficient value of 0.72 is an indication that HEC-HMS streamflow has an acceptable covariance with the observed flow but not to the extent of WRF-Hydro covariance which is higher than 0.9. The PBias value of − 32.47% indicates that the model systematically underestimates the observed hydrograph volume after the rising limb. In comparison to the WRF-Hydro findings, performance metrics including RMSE, KGE, and NSE that measure the overall fit of the simulated hydrograph demonstrate a lower performance for the HEC-HMS model (Table 3).

It is important to note that the HEC-HMS model in its operational configuration is run continuously. In addition, the model is forced with an ensemble of around 100 members that include GEFS, ECMWF, and NARR precipitation estimates. The use of NLDAS in this study to remain consistent with WRF-Hydro may have introduced a discrepancy between the obtained results with an event-based run with HEC-HMS and those generated, in 2011, with the continuous operational version of HEC-HMS which were reported in Saleh et al. (2017). Nevertheless, the obtained streamflow with HEC-HMS in this study is comparable to those reported in Saleh et al. (2017), especially with the forecast generated on August 26, 2011, where most of the members showed an underestimation of the observed streamflow as reported in this study. The peak flow values of ensemble forecasts with different lead times (24, 48, and 72 h) at New Milford station (01378500) range between 20 and 525 m^3^/s. Validation results show that the peak flow simulated by WRF-Hydro stays within the range of those simulated with the SFAS flood advisory system. In terms of peak flow time, the forecast members have a lag of up to 24 h, whereas WRF-Hydro has a 1-hour delay relative to the observed peak timing.

The assessment of WRF-Hydro simulated streamflow values during Hurricane Irene and those obtained during the same event with HEC-HMS in the SFAS system indicates that WRF-Hydro has the potential to be integrated into the operational flood system. In this regard, WRF-Hydro offers an improvement over the lumped version of the HEC-HMS model. In this study, WRF-Hydro with its gridded configuration of the surface routing can simulate at the 50-m pixel level overland flow which allows it to capture the spatial distribution of flood inundation. This said, a more advanced calibration procedure that accounts for all existing USGS stations within the watershed remains necessary and should lead to an enhancement of all sub-watersheds rather than optimizing the watershed-wide parameters to achieve the performance goals at the most downstream station.

### Evaluation of high-water marks (HWM)

**Fig. 5:**
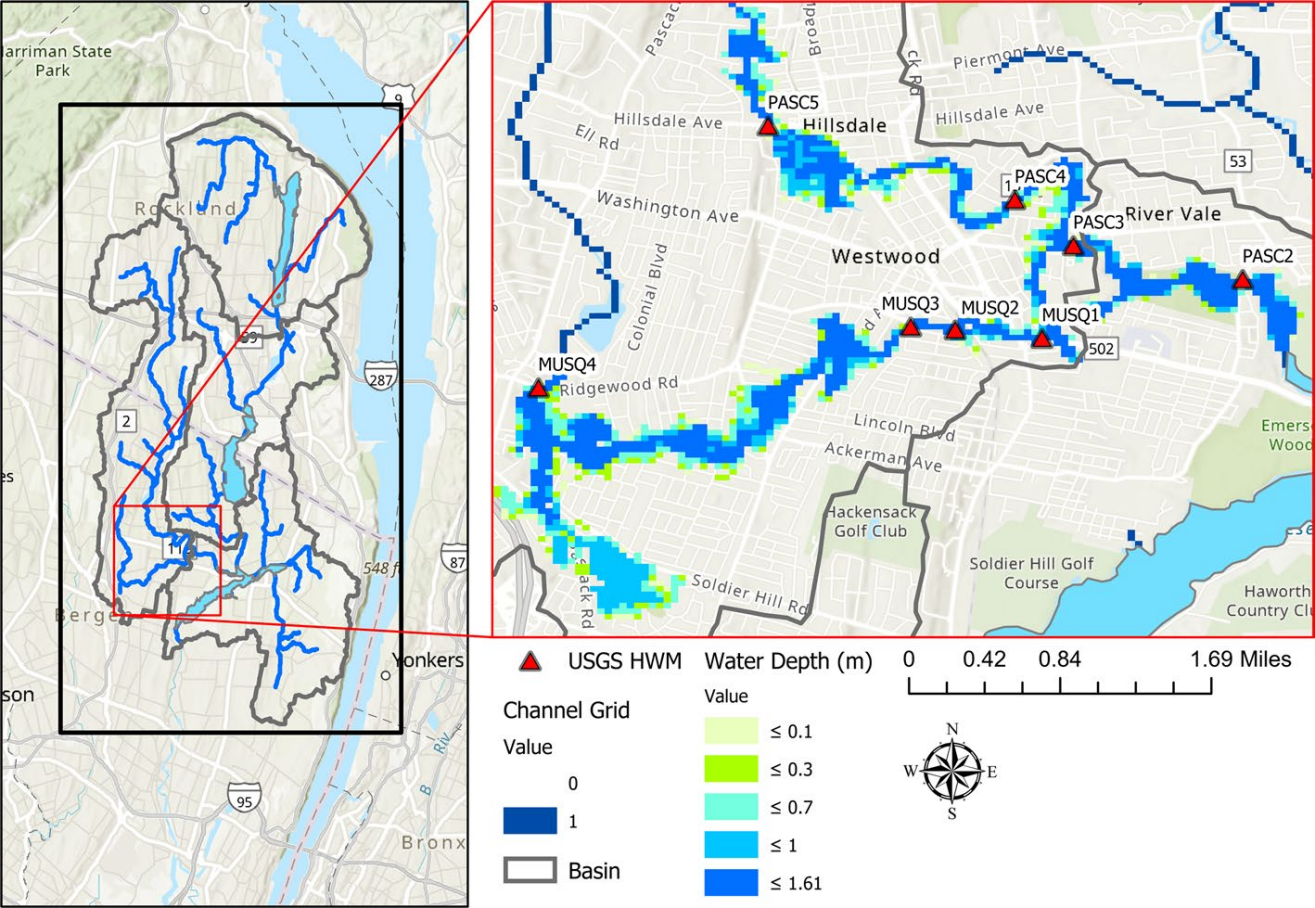
Evaluation of the WRF-Hydro inundation depth generated using the HAND method against the surveyed HWMs

**Fig. 6:**
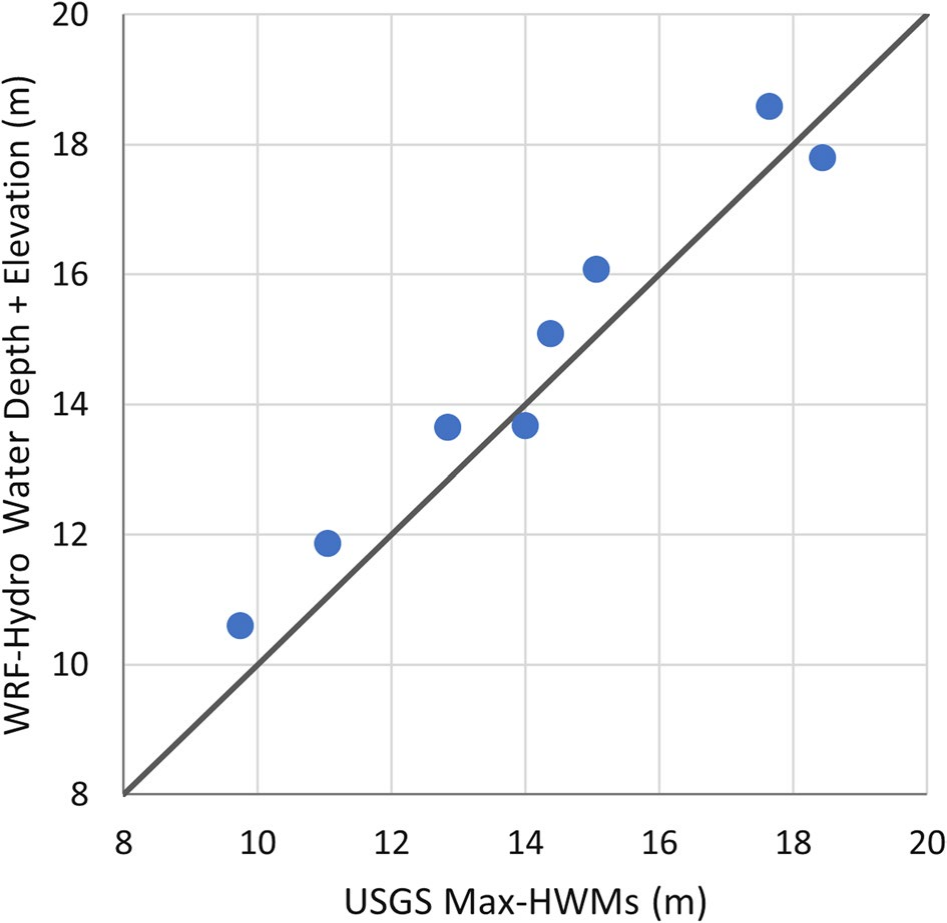
Comparison of surveyed HWMs and the simulated water level (WRF-Hydro water depth + elevation)

USGS reported eight HWMs reported by USGS in the southern part of the watersheds in the aftermath of Hurricane Irene (August 28, 2011). These HWMs were compared to simulated water level values inferred from the peak streamflow simulated using WRF-Hydro. USGS reported HWM along the Hackensack River tributaries Pascack and Musquapsink Brooks as shown in Fig. 5. The used water depth values from WRF-Hydro were inferred from the simulated streamflow using SRCs. The HAND method was used to generate the flood inundation maps in Fig. 5. The extent of flood inundation in Fig. 5 varied along the Hackensack River and its tributaries. This can be attributed to changes in topography and the used SRCs which may reflect a change in the river cross-section. Figure 6 shows the scatter plot for the comparison of the 8 HWMs and the estimated water level from WRF-Hydro at the same locations. The PBias was limited to 4%, the RMSE was equal to 0.8 m, and the MAE was 0.78 m. It is important to add that the location of the HWM sampling points in proximity to the river and within its floodplain may have contributed to the obtained agreement between the observed and simulated streamflow values. Still, the reported discrepancy could be attributed to the inaccuracy in the determination of HWMs vertically and horizontally, namely, the water depth and the exact location of points, respectively. In this study, the water depths from WRF-Hydro were inferred from the inundated grid cell that is assumed to be collocated with the surveyed HWM. Considering a larger parcel and calculating the representative model water depths from estimates like the maximum, minimum, and areal averaged water depth values as suggested by Smith et al. (2021) can contribute to improving the agreement between the surveyed and simulated HWM.

### Comparison of inundation extents

**Fig. 7:**
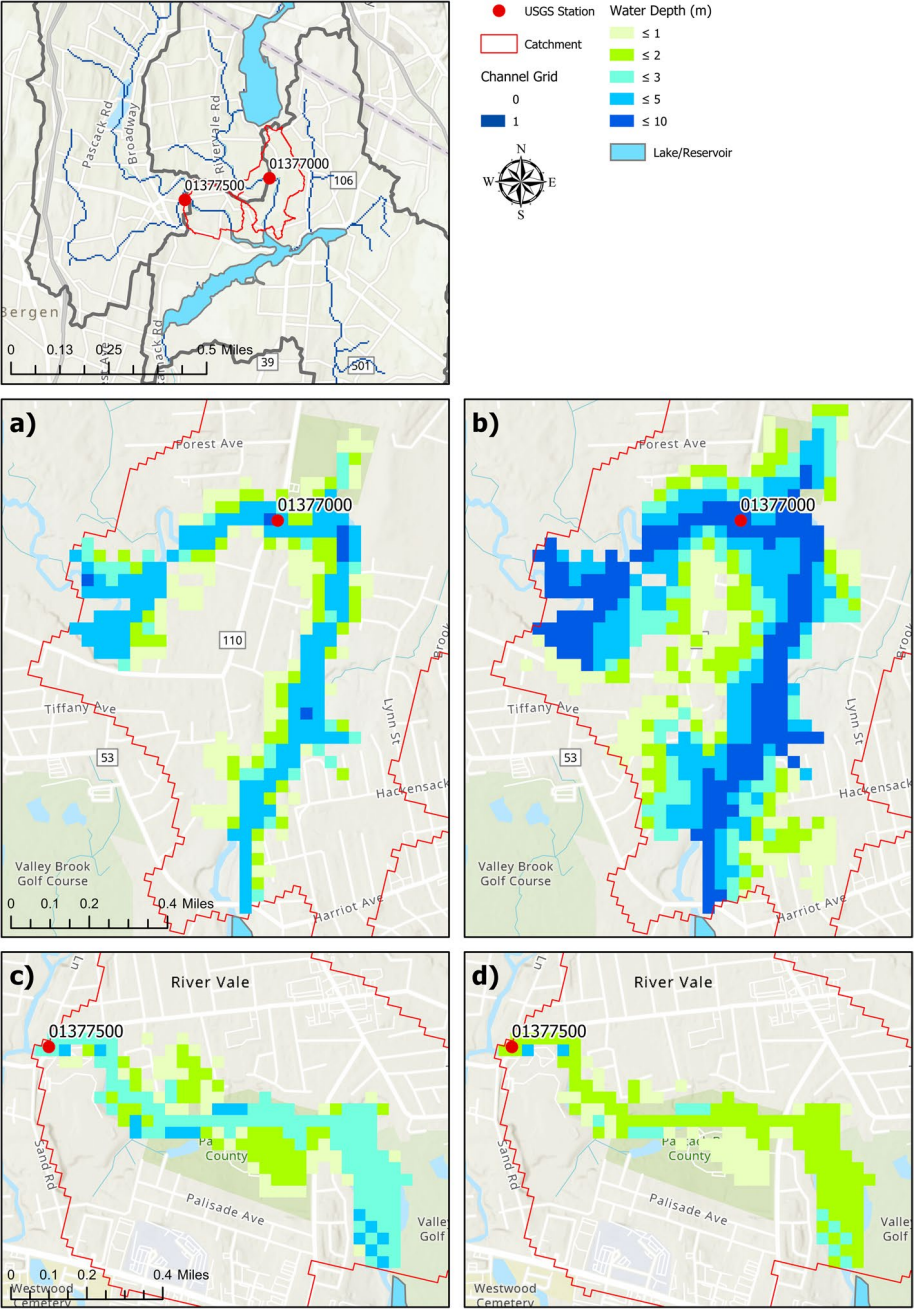
Inundation depths generated with the HAND method by using USGS (**a** and **c**) and WRF-Hydro maximum stage (**b** and **d**) values. The red line represents the sub-catchment of specified NWM reaches in the NHDPlus V2.1 dataset

Figure 7 compares the flood inundation extent derived by using the HAND method based on the water level values from USGS station records and the WRF-Hydro model for the sub-catchments obtained from NHDPlus V2.1 dataset corresponding to the stations (01377000 and 01377500). The water level value from the WRF-Hydro is determined by following the same approach used in Fig. 5. Flood inundation extents based on the USGS water level records (Fig. 7a, c) are considered as the benchmark extents compared to the WRF-Hydro flood inundation extents (Fig. 7b, d). Table 4 shows the surface area values for inundation extent generated with the peak water level values obtained from USGS records and WRF-Hydro simulation. For the sub-catchment corresponding to station-01377000, the inundation extent area of WRF-Hydro captures nearly twice that of the USGS as shown in Figs. 7a and b. This result is in line with the difference noticed between the observed and the simulated water levels (Table 4) where WRF-Hydro reported a water level that is higher than the observed one. In contrast, the inundation extent of WRF-Hydro underestimated the extent of the inundated area that is covered by the USGS inundation extent for station-01377500 (Fig. 7c, d). The discrepancies between the generated maps can be attributed to the difference in the channel geometries as WRF-Hydro uses an idealized trapezoidal channel which does not necessarily match the actual river cross section that is monitored by USGS.

**Table 4:** Simulated peak water depth within the sub-watersheds and the inundation extent based on observed (USGS) and simulated (WRF-Hydro) peak water depth values

Station	USGS		WRF-Hydro	
	Peak water depth (m)	Surface area (10^3^ m^2^)	Peak water depth (m)	Surface area (10^3^ m^2^)
01377000	3.74	747.5	6.56	1410
01377500	2.65	502.5	1.58	377.5

### Evaluation of crowdsourced data

**Fig. 8:**
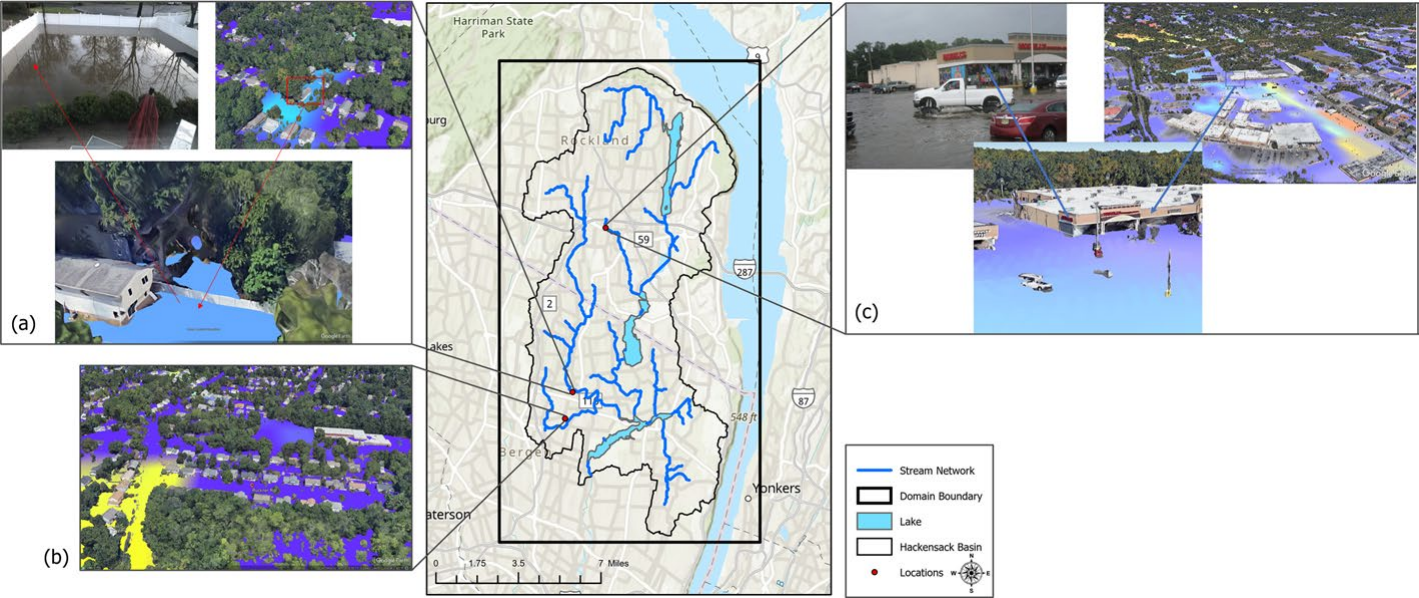
Qualitative evaluation of WRF-Hydro surface head outputs with geotagged crowdsourced data

The crowdsourced information for the remnants of Storm Ida is obtained from the residents of the towns in the studied domain and the Office of Emergency Management (OEM) of the town of Westwood, NJ, which is located in the southwest of the watershed, downstream of the Woodcliff Lake Reservoir that is part of the Hackensack watershed reservoirs system. For a qualitative assessment of the model, the geotagged photographs taken after the events were used. Then, the maximum surface head output generated by the WRF-Hydro model for Storm Ida is compared to the geotagged images in Fig. 8. Based on an image taken by a resident on Harding Avenue in Westwood, NJ, it was observed that the backyard of a house was flooded up to 1/5 of the height of the fences, which are typically 6 feet in height (Fig. 8a). This water depth corresponds to ~ 365 mm. On the other hand, the WRF-Hydro surface head results for Storm Ida, show that the same location is inundated with a water depth of around 60 mm. The comparison between the two sources shows that the WRF-Hydro model significantly underestimates the actual water depth. Despite this underestimation, the model was still able to accurately predict the occurrence of flooding in the reported area.

According to the water depth data collected on Ruckner Rd, Westwood, NJ, the water level reached up to 6.4 inches (~ 163 mm). Similarly, the findings from WRF-Hydro indicate that the same location experiences inundation with a water depth of 193 mm (~ 7.6 inches) (Fig. 8b).

The same comparison is made for an image taken at a marketplace in Nanuet, NY, after Hurricane Irene for the qualitative assessment of the model (Fig. 8c). The image is a representative example of several other reports and images published by local media in the aftermath of Hurricane Irene like (2011b). This qualitative analysis also highlights that the model, based on the visual inspection, is capable of approximating the water depth seen in the images taken right after Hurricane Irene. Nevertheless, from the crowdsourced picture, despite the qualitative nature of the information, one can state that the water depth at the surveyed location is between 1 and 2 inches by looking at the white truck and the water level that is reaching almost half of the tire. At the same location, the examination of the WRF-Hydro map reports a water depth in the order of one inch or lower. The used images, like many others, reported by the local media stream can be geotagged manually. However, most of them are not time-stamped, which may introduce a discrepancy between the conditions they report and those corresponding to the peak flow in WRF-Hydro. Future work will focus on automating the process of gathering crowdsourced images, geotagging, and processing them to extract quantitative information that can be useful for the calibration and validation of WRF-Hydro with an eventual integration in the National Water Model. Future work can also cover the investigation of leveraging social media using Natural Language Processing (NLP) techniques to infer information on extreme weather events and their impacts that could be useful to calibrate and validate hydrological models (Tounsi and Temimi 2023).

## Discussions

The goal behind using the WRF-Hydro model at high resolution with its gridded configuration is to develop the capability to simulate flood inundation at block scale in a densely populated watershed in the operational Stevens Flood Advisory System. The obtained results showed an acceptable performance of the model to simulate such conditions which makes WRF-Hydro a suitable candidate for an eventual integration in the SFAS system to replace the lumped HEC-HMS model. Nevertheless, it is desirable to generate urban flood inundation maps at higher resolution in the order of 10 m. Previous studies were able to successfully deploy WRF-Hydro at higher resolution (Kim et al. 2021; Smith et al. 2021). In this study, various other configurations of WRF-Hydro were tested (not reported here) before selecting the one used in this study. The simulations at higher resolution led to a significant instability of the model, despite the adjustment of the timesteps for the channel and overland routing in the namelist. This could be attributed to the topographic gradient in the Hackensack watershed which generates a steep slope in the northwestern area of the watershed whereas the northern and eastern parts have a gentle slope. The other possible reason is the existence of several lakes and reservoirs cascading water from the most upstream northern section of the watershed to the downstream station at New Milford, right after the Oradell reservoir. The actual configuration of the reservoirs in the watershed involves bypasses that divert the water to the stream parallel to the Hackensack River in case of an overtopping. This behavior is still not possible to capture in the current version of WRF-Hydro which is limited to the modeling of the orifice and weir, the latter for overtopping and the former for the release mechanism. The lack of capability to account for water control management poses a constraint on streamflow prediction over the regulated river systems (Kim et al. 2020). Not being able to capture the bypassing process may hamper the calibration processes and the determined parameter sets as observed in this study. Future work may cover the introduction of the capability to better control streamflow released by lakes and reservoirs by accounting for operation rules. One method that can be considered is to introduce the use of a machine learning-based method to capture reservoirs’ operational rules (Tounsi et al. 2022) and embed them in WRF-Hydro.

The modeling of lakes and reservoirs in WRF-Hydro is somehow basic, unlike the modeling options that are available in HEC-HMS which allow the user to account for the storage-discharge function and the use of elevation-area relationship as a rating curve specific to the reservoir. As observed in this study area, the Hackensack watershed comprises a set of reservoirs that cascade the discharge towards the basin outlet at the New Milford station and so does the error in the simulation of reservoir releases. Therefore, the simplicity of the modeling of the behavior of reservoirs in WRF-Hydro can introduce an added uncertainty in the modeling of the discharge in the watershed.

The version of HEC-HMS that is used operationally in the SFAS system does not permit the simulation of the gridded runoff. The recent releases of HEC-HMS (version 4.7 onward) started to allow the use of gridded configurations through the processing of terrain information in the model instead of using HEC-GeoHMS separately (U.S. Army Corps of Engineers 2021). Nevertheless, the channel routing is still conducted using the hydrologic routing options and the use of hydraulic routing requires another model like HEC-RAS. Moreover, WRF-Hydro is an open-source model that gives the user the capability of adjusting the schemes in the models and their formulation. On the other hand, other common models in hydrology like HEC-HMS are free but disseminated in compiled form that does not give the user the possibility to change their codes and models’ formulations. LISFLOOD-FP, specializing in floodplain inundation modeling, could be a viable alternative to WRF-Hydro, especially with its capability to model pluvial and fluvial processes. However, with the intention to eventually integrate the WRF-Hydro model into the National Water Model which uses WRF-Hydro in its engine, WRF-Hydro was selected in this study for consistency reasons. In addition, WRF-Hydro can be used as a two-way coupled model (although not adopted in this study) where the hydrologic and atmospheric processes are coupled for the simulation of the land–atmosphere interactions and the feedback of soil moisture to precipitation after its spatial redistribution due to runoff.

Another possible improvement of the modeling urban-scale processes with WRF-Hydro can cover the quality of the topographic information that is ingested by the model. Additionally, the capability of simulating overland flow at high resolution makes the WRF-Hydro well-suited for capturing the small-scale variations on the terrain and land cover impacting the hydrological and hydraulic processes. This capability holds a particular importance over urbanized watersheds like the Hackensack watershed. Besides that, the coupling of overland and channel routing components helps to generate more realistic conditions, especially in scenarios where both fluvial- and pluvial-sourced flood events are observed. However, there is no two-way coupling between overland flow and overbank flooding. Consequently, volumes from overflows are virtually accounted for in the model to maintain mass conservation without impacting overland routing hydraulically. A post-processing step is needed to convert the volume of the overbank flood into inundation maps using various methods, such as the Height Above Nearest Drainage (HAND) method employed in this study. However, this step might introduce additional errors due to the synthetic rating curves (SRCs) utilized to convert streamflow to stage value (Johnson et al. 2019).

While WRF-Hydro considers the urban land cover characteristics at a high-resolution level, it does not account for the impact of urban infrastructures like levees, pumping stations, minor drainage networks, or conduits. This limitation can lead to missing the backwater effect due to these hydraulic structures or uncertainties sourced from missing urban drainage representation within the modeled urban hydrological system. It is also critical in an urban area to properly capture the obstruction introduced by the existing buildings and infrastructures. Even though the original DEM that was used in the WRF-Hydro Pre-processing Toolbox in ArcGIS is of high resolution as it was developed from LIDAR flyovers, it remains that a significant part of the topographic details is lost when aggregating the information to match the overland flow grid scale (50-m in this study). Future work should consider refining the DEM by exaggerating the building footprint to achieve a better-defined surface flow drainage network that captures the flow in street gutters as well as the obstructions imposed by buildings and existing infrastructures which is very important to assess the actual impact on infrastructure.

The NOAA Office of Water Prediction (OWP) launched the National Water Model (NWM) in 2016, leveraging the WRF-Hydro model. Through continued investments, the NWM has been expanded to generate streamflow outputs for over 2.7 million points seamlessly over CONUS. In 2024, OWP is planning to switch to a model agnostic modeling framework called Next Generation (Nextgen) NWM. Within this framework, users will have the capability to integrate specific model formulations tailored to regional streamflow generation processes. This provides enhanced flexibility to deploy diverse models or schemes within the same watershed of interest. Notably, the WRF-Hydro, serving as the current NWM engine, stands as one of the candidate models intended for integration into this framework. This presents a distinct advantage for WRF-Hydro for integration as a high-resolution model within the Nextgen framework.

## Conclusions

This study addressed the deployment of the WRF-Hydro model to simulate streamflow and flood inundation in the Hackensack River Watershed, in the New York City metropolitan area. The model was configured to run with a gridded configuration where the land surface model has a 200-m resolution and the routing model for surface and channel has a 50-m resolution. The calibration of the model involved the adjustment of hydrologic and hydraulic parameters. Finally, the model was validated using the extreme event of Hurricane Irene, in August 2011. The model results were compared to (a) streamflow observations, (b) the simulated streamflow values using the default version of WRF-Hydro, and (c) the outcome of the HEC-HMS model that is used in the operational Stevens Flood Advisory System. The analysis of the results revealed that the calibrated version of the WRF-Hydro model led to a stronger agreement with the observed streamflow. Nevertheless, both the calibrated and default versions of the model exhibited limitations in the modeling of baseflow within the watershed. In addition, High Water Marks, which were reported by USGS, as an indicator of the maximum water level in the aftermath of the event showed that the model accurately simulated the water depth. The flood extents simulated with WRF-Hydro were either larger or smaller than the inferred extent from USGS water level observations. This difference can be attributed to the use of synthetic rating curves which might be inaccurate in converting the simulated streamflow with WRF-Hydro to flood inundation along with the mentioned discrepancies in streamflow simulations sourced from the model itself. Future work should focus on enhancing WRF-Hydro capabilities to simulate flood inundation online with more reliable rating curves without the need to infer flood inundation offline as a postprocessing step.
